# Calculation of the pH Values of Aqueous Systems Containing Carbonic Acid and Significance for Natural Waters, Following (Near-)Exact and Approximated Solutions: The Importance of the Boundary Conditions

**DOI:** 10.3390/molecules31020292

**Published:** 2026-01-14

**Authors:** Arianna Rosso, Davide Vione

**Affiliations:** Dipartimento di Chimica, Università degli Studi di Torino, Via Pietro Giuria 5, 10125 Torino, Italy; arianna.rosso336@edu.unito.it

**Keywords:** carbonate chemistry, equilibrium calculations, charge balance, numerical solutions of higher-order equations, alkalinity, Python coding

## Abstract

Calculating the pH values of carbonic acid solutions is an important task in studies of chemical equilibria in freshwater systems, with applications to environmental chemistry, geology, and hydrology. These pH values are also highly relevant in the context of climate change, since increasing atmospheric CO_2_ affects the concentration of dissolved carbon dioxide and carbonic acid, collectively denoted as [H_2_CO_3_*] = [H_2_CO_3(aq)_] + [CO_2(aq)_]. Solving equilibrium systems to obtain analytical functions is particularly useful when such functions are required, for example, in data fitting. We show here that, although exact or near-exact solutions typically result in third- to fourth-order equations that must be solved numerically, reasonable approximations can be derived that lead to analytical second-order equations. In this framework, the chosen approximations need to meet the boundary conditions of the systems, particularly for c_T_ → 0 and for high c_T_ values (where c_T_ = [H_2_CO_3_*] + [HCO_3_^−^] + [CO_3_^2−^]). Finally, we provide exact solutions for a closed system containing both H_2_CO_3_* and alkalinity, which enables the description of virtually any aquatic environment without assuming equilibrium with atmospheric CO_2_. Implications for pH calculations in natural waters are also briefly discussed.

## 1. Introduction

The carbonate system is a key component in natural waters, where it exerts primary control on pH. In many cases, the pH of these systems can be interpreted as the outcome of a titration process between carbonate minerals and atmospheric CO_2_. For instance, in the case of dolomite, the governing acid–base reaction can be expressed as follows [1] (note that the subscript _(g)_ refers to the gas phase, _(s)_ to solid, _(l)_ to liquid, and _(aq)_ to water-dissolved species): 

CaMg(CO_3_)_2 (s)_ + 2 CO_2 (g)_ + 2 H_2_O _(l)_ ⇆ Ca^2+^_(aq)_ + Mg^2+^_(aq)_ + 4 HCO_3_^−^_(aq)_ (1)

Such a process is clearly affected by the anthropogenic emissions of carbon dioxide, and the resulting impact on the pH values of natural surface waters is often called “the other side effect of climate change”. Ocean acidification is the best known effect of the increase in atmospheric CO_2_ levels [2], but similar processes are also observed in natural surface waters. For instance, a long-term study into the chemistry of river waters in Switzerland has yielded the following [1]:

(i). Reaction (1) is responsible for the long-term increase in Mg levels;
(ii). Contrary to Mg, Ca levels rather underwent a long-term decrease. The most likely reason is that, differently from MgCO_3_, CaCO_3_ is very near saturation in Swiss rivers and the increase in water temperature has caused a decrease in Ca^2+^ solubility.

For the above reasons, the actual reactions occurring in Swiss rivers and accounting for the observed concentration trends are presumed to be the following (in Equation (3), “Δ” means “heat”) [1]:

CaMg(CO_3_)_2(s)_ + CO_2(g)_ + H_2_O_(l)_ ⇆ CaCO_3(s)_ + Mg^2+^_(aq)_ + 2 HCO_3_^−^_(aq)_ (2)

(3) $${\mathrm{Ca}}_{\left(\mathrm{aq}\right)}^{2+}+{{\mathrm{CO}}_{3}}_{\left(\mathrm{aq}\right)}^{2-}\;\overset{\Delta }{\to }{\mathrm{CaCO}}_{3(s)}$$

The calculation of the pH values in natural waters, based on equilibrium reactions involving carbonate species, plays an understandably important role in several fields, including environmental chemistry, hydrology, and climate change science. This issue can be addressed through different approaches.

One possibility is the numerical solution of equation systems arising from multiple equilibria, using dedicated software packages developed for the description of surface waters, groundwater [3], seawater [4] and, more generally, laboratory solutions designed for the study of chemical equilibria [5]. These numerical approaches have the considerable advantage of being highly accurate. However, the relevant software packages often operate as “black boxes”. Moreover, the exclusive reliance on numerical solutions prevents the derivation of parameter-adjustable equations. Such equations may be of particular importance when fitting experimental or environmental data, or when a rapid evaluation of the effect of varying parameters on equilibrium pH values is required [6]. To achieve a similar outcome with software packages, multiple runs are necessary that may become impractical, especially when a high resolution is desired. In such cases, an extremely large number of repeated simulations with only slightly modified input parameters would be needed.

In this work, we address the problem of calculating pH values in carbonate systems using chemical equilibria, along with mass and charge balances, to derive analytical equations. To maintain the highest degree of generality, we adopt a closed-system description, avoiding the alternative open-system approach. The rationale is that, while the open-system description simplifies the mathematics, it assumes that natural waters are in equilibrium with the dissolution of atmospheric CO_2_, which is not always the case in environmental aquatic systems [7].

First, we provide (near-)exact solutions to the equilibrium calculations, which, however, result in third- to fourth-order equations that must be solved numerically. Still, with the aid of suitable tools (e.g., Python-based codes [8]), it becomes relatively straightforward to assess how changes in parameters such as total carbonate concentration or alkalinity affect the equilibrium pH.

Whenever possible, we also provide more manageable approximate solutions based on second-order equations. Comparing the exact (or near-exact) and approximate solutions, allows us to identify the essential features that an approximate solution must possess to reliably describe the relevant system, across a broad range of conditions. In particular, we highlight the critical importance to satisfy the boundary conditions of the system, as they strongly influence the behavior of the approximate solutions.

The conclusions of this study have significant implications for the calculation of the pH values of natural waters that contain inorganic carbon species.

## 2. Results and Discussion

Carbonic acid (H_2_CO_3(aq)_) is one of the dissolved forms in which carbon dioxide occurs in an aqueous solution, the other species being CO_2(aq)_. To avoid the complication of treating the H_2_CO_3_/CO_2_ equilibrium on top of all the others, the fictitious species H_2_CO_3_* is often introduced. [H_2_CO_3_*] is defined as concentration sum of H_2_CO_3(aq)_ and CO_2(aq)_. In this framework, H_2_CO_3_* contributes the same amount of H_3_O^+^ and HCO_3_^−^ as H_2_CO_3(aq)_, noting that CO_2(aq)_ does not participate in acid-base reactions [9].

CO_2(g)_ ⇆ CO_2(aq)_ (4)

CO_2(aq)_ + H_2_O_(l)_ ⇆ H_2_CO_3(aq)_ (5)

[H_2_CO_3_*] = [CO_2(aq)_] + [H_2_CO_3(aq)_], with [CO_2(aq)_] » [H_2_CO_3(aq)_] (6)

Considering an aqueous solution that initially contains H_2_CO_3_* at a total concentration c_T_, the following equilibria are established, with equilibrium constants K_a1_* = 5 × 10^−7^ and K_a2_ = 5 × 10^−11^. Note that K_a1_* ≈ 1.5 × 10^−3^ K_a1_, because K_a1_* also takes into account the equilibrium reaction (5). Moreover, K_w_ is the self-protolysis constant of water [10].

(7) $$H_{2}{\mathrm{CO}}_{3}*+H_{2}O\;⇆\;H_{3}O^{+}+{{\mathrm{HCO}}_{3}}^{-}\;\;\;\;\;\;\;\;\;K_{a1}^{*}=\frac{{[H}_{3}O^{+}]\;{[\mathrm{HCO}}_{3}^{-}]}{{[H}_{2}{\mathrm{CO}}_{3}^{*}]}=10^{-6.3}$$

(8) $${{\mathrm{HCO}}_{3}}^{-}+H_{2}O\;⇆\;H_{3}O^{+}+{{\mathrm{CO}}_{3}}^{2^{-}}\;\;\;\;\;\;\;\;K_{a2}=\frac{{[H}_{3}O^{+}]\;{[\mathrm{CO}}_{3}^{2-}]}{{[\mathrm{HCO}}_{3}^{-}]}=10^{-10.3}$$

2 H_2_O ⇆ H_3_O^+^ + OH^−^       K_w_ = [H_3_O^+^] [OH^−^] = 10^−14^ (9)

c_T_ = [H_2_CO_3_*] + [HCO_3_^−^] + [CO_3_^2−^] (10)

Equations (7)–(10) form a system, which can be solved to yield the following expressions for the fraction of each species (α_0_ for H_2_CO_3_*, α_1_ for HCO_3_^−^, and α_2_ for CO_3_^2−^):

α_0_ = [H_2_CO_3_*]/c_T_ = [H_3_O^+^]^2^ ([H_3_O^+^]^2^ + K_a1_* [H_3_O^+^] + K_a1_* K_a2_)^−1^ (11)

α_1_ = [HCO_3_^−^]/c_T_ = K_a1_* [H_3_O^+^] ([H_3_O^+^]^2^ + K_a1_* [H_3_O^+^] + K_a1_* K_a2_)^−1^ (12)

α_2_ = [CO_3_^2−^]/c_T_ = K_a1_* K_a2_ ([H_3_O^+^]^2^ + K_a1_* [H_3_O^+^] + K_a1_* K_a2_)^−1^ (13)

**Figure 1:**
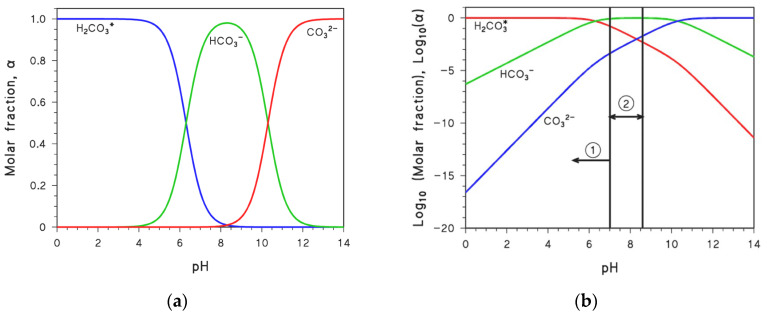
(**a**) pH trend of the carbonate species fractions. (**b**) pH trend of the fraction logarithms. Two pH intervals are highlighted: ① Boundary conditions for a solution of H_2_CO_3_*, and ② boundary conditions for a solution containing Ca^2+^ and HCO_3_^−^.

Figure 1 reports the trends of α_0–2_ (Figure 1a), as well as of Log_10_(α_0–2_) (Figure 1b), as a function of pH. The pH intervals that are relevant to a solution containing H_2_CO_3_*, and to a system based on Ca^2+^ + HCO_3_^−^ (vide infra), are also highlighted in Figure 1b.

### 2.1. Calculation of the pH Value of a Solution of H_2_CO_3_*, Having Total Concentration c_T_

Such a system has the following, well-known boundary conditions [7,9]:

(a) If c_T_ is high enough, then

(14) $$[H_{3}O^{+}]=\sqrt{K_{a1}^{*}c_{T}}$$

(b) If c_T_ → 0, then [H_3_O^+^] = (K_w_)^½^ (typically, pH 7)

The equilibria in this system are described by reactions (7)–(9). From the charge balance, it follows that the sum of the concentrations of cations ([H_3_O^+^]), weighted by their charge, is equal to the sum of the concentrations of the anions (HCO_3_^−^, CO_3_^2−^, OH^−^), also weighted by their charge:

[H_3_O^+^] = [HCO_3_^−^] + 2 [CO_3_^2−^] + [OH^−^] (15)

Very often, to solve the charge balance of a solution of a diprotic acid, it is suggested to neglect [OH^−^], which might be reasonable in an acidic solution [7,9]. However, such an approximation would be in contrast with the boundary condition (b), which is valid if c_T_ → 0. By neglecting [OH^−^], the approximated solution would thus lose generality.

In contrast, it would be much safer to neglect [CO_3_^2−^]. In fact, in a solution containing H_2_CO_3_*, the pH is expected to be ≤7, thus [CO_3_^2−^] < 10^−3^ [HCO_3_^−^] (see Equation (8) and Figure 1). Therefore, Equation (15) can be simplified as follows, without significant loss of generality or accuracy:

[H_3_O^+^] = [HCO_3_^−^] + [OH^−^] (16)

By application of Equations (9) and (12), Equation (16) becomes the following:

(17) $${[H}_{3}O^{+}]=\frac{K_{a1}^{*}{[H}_{3}O^{+}]}{{{[H}_{3}O^{+}]}^{2}+K_{a1}^{*}{[H}_{3}O^{+}]+K_{a1}^{*}K_{a2}}c_{T}+\frac{K_{w}}{{[H}_{3}O^{+}]}$$

At the denominator of the first right-hand term of Equation (17), it is found that [H_3_O^+^]^2^ is proportional to [H_2_CO_3_*], K_a1_* [H_3_O^+^] is proportional to [HCO_3_^−^], and K_a1_* K_a2_ is proportional to [CO_3_^2−^] (see Equations (11)–(13)). Therefore, [CO_3_^2−^] « [HCO_3_^−^] implies K_a1_* K_a2_ « K_a1_* [H_3_O^+^]. By so doing, Equation (17) simplifies as follows:

(18) $${[H}_{3}O^{+}]=\frac{K_{a1}^{*}{[H}_{3}O^{+}]}{{{[H}_{3}O^{+}]}^{2}+K_{a1}^{*}{[H}_{3}O^{+}]}c_{T}+\frac{K_{w}}{{[H}_{3}O^{+}]}=\frac{K_{a1}^{*}}{{[H}_{3}O^{+}]+K_{a1}^{*}}c_{T}+\frac{K_{w}}{{[H}_{3}O^{+}]}$$

In other words, by neglecting [CO_3_^2−^] and K_a1_* K_a2_, the case of H_2_CO_3_* reduces to a monoprotic acid problem. Because the approximations made so far are well justified, we get a near-exact solution of the problem in the form of a cubic equation that reads as follows:

(19) $${{[H}_{3}O^{+}]}^{3}+K_{a1}^{*}{{[H}_{3}O^{+}]}^{2}-(K_{a1}^{*}c_{T}+K_{w}{)[H}_{3}O^{+}]-K_{a1}^{*}K_{w}=0$$

Equation (19) can be solved either analytically or numerically. We solved it numerically using the “fsolve” command in Python, which solves non-linear equations by using an iterative procedure (see the Section 3 for the complete Python script) [11].

**Figure 2:**
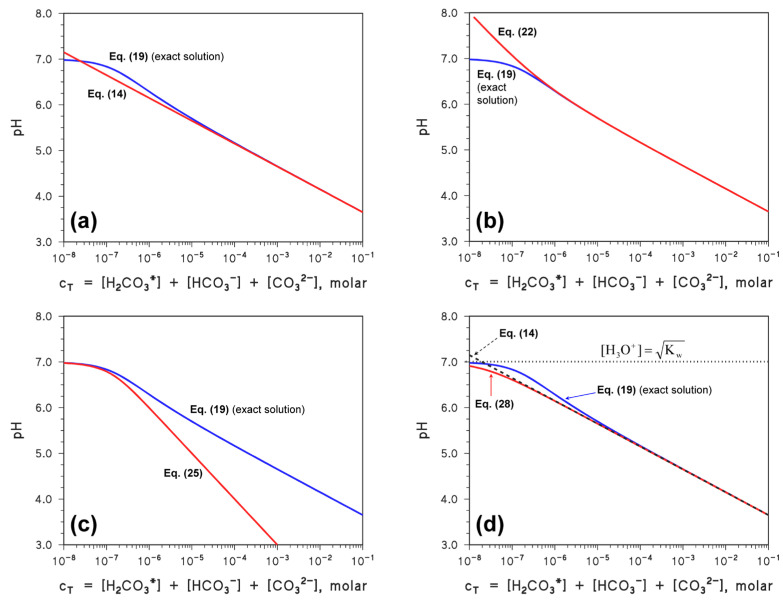
Plots (pH vs. c_T_) of the exact solution to Equation (19) and of approximated solutions: (**a**) Equation (14); (**b**) Equation (22); (**c**) Equation (25), and (**d**) Equation (28) (see the main text to get insight into the relevant approximations).

By so doing, we obtained the results shown as “exact solution” in Figure 2. Equations (16) and (18) can be further simplified under the hypothesis that [HCO_3_^−^] » [OH^−^]. This condition holds when c_T_ is sufficiently high, which means that there is significant impact of the deprotonation of H_2_CO_3_* on the solution pH (in other words, deprotonation of H_2_CO_3_* acidifies the solution to a significant degree). With these premises, Equation (18) reduces to:

(20) $${[H}_{3}O^{+}]=\frac{K_{a1}^{*}}{{[H}_{3}O^{+}]+K_{a1}^{*}}c_{T}$$

The result is a second-order equation:

(21) $${{[H}_{3}O^{+}]}^{2}+K_{a1}^{*}{[H}_{3}O^{+}]-K_{a1}^{*}c_{T}=0$$

The solution of Equation (21) is:

(22) $${[H}_{3}O^{+}]=\frac{-K_{a1}^{*}+\sqrt{{\left(K_{a1}^{*}\right)}^{2}+4K_{a1}^{*}c_{T}}}{2}$$

The extent to which Equation (22) approximates the exact solution is shown in Figure 2b. It can be seen that the agreement is excellent for c_T_ > 10^−6^ M. Another possible approximation for Equation (18) is K_a1_* » [H_3_O^+^], which yields:

(23) $${[H}_{3}O^{+}]=c_{T}+\frac{K_{w}}{{[H}_{3}O^{+}]}$$

Equation (23) means that the concentration of H_2_CO_3_* is low enough that the acid behaves as a strong monoprotic one, undergoing complete deprotonation ([HCO_3_^−^] = c_T_). Such a contribution adds to water self-protolysis, represented by the term K_w_ [H_3_O^+^]^−1^. The approximation thus made yields the following second-order equation:

(24) $${{[H}_{3}O^{+}]}^{2}-c_{T}{[H}_{3}O^{+}]-K_{w}=0$$

(25) $${[H}_{3}O^{+}]=\frac{c_{T}+\sqrt{{\left(c_{T}\right)}^{2}+4K_{w}}}{2}$$

Equation (25) is shown in Figure 2c, indicating that the approximation is excellent if c_T_ < 10^−7^ M. This result is consistent with the low-c_T_ assumption underlying Equations (23)–(25).

Finally, if K_a1_* « [H_3_O^+^] (the opposite condition to that above), Equation (18) simplifies as follows:

(26) $${[H}_{3}O^{+}]=\frac{K_{a1}^{*}}{{[H}_{3}O^{+}]}c_{T}+\frac{K_{w}}{{[H}_{3}O^{+}]}$$

(27) $${{[H}_{3}O^{+}]}^{2}-K_{a1}^{*}c_{T}-K_{w}=0$$

This very simple second-order equation has the following solution:

(28) $${[H}_{3}O^{+}]=\sqrt{K_{a1}^{*}c_{T}+K_{w}}$$

Very interestingly, Equation (28) thoroughly meets the boundary conditions specified at the beginning of this section, namely $\underset{c_{T}\to 0}{\lim}$[H_3_O^+^] = $\sqrt{K_{w}}$ and $\underset{\mathrm{High}\;\;c_{T}}{\lim}$ [H_3_O^+^] = $\sqrt{K_{a_{1}}^{*}c_{T}}$.

Notably, Equation (28) provides a close approximation to the exact solution (Figure 2d), except in the range where the assumption K_a1_* « [H_3_O^+^] breaks down (K_a1_* >≈ [H_3_O^+^]). However, even in these circumstances, there are conditions (c_T_ → 0) where [OH^−^] » [HCO_3_^−^]. In this regime, the approximation K_a1_* « [H_3_O^+^] for [HCO_3_^−^] is no longer significant.

From the above discussion, it is apparent that the approximation that best matches the exact solution across the entire c_T_ range (Equation (28)) is also the only one that fully satisfies the boundary conditions at the extremes of the c_T_ variation range. This finding suggests that the relatively simple Equation (28) is very suitable to calculate the pH of a H_2_CO_3_* solution.

### 2.2. Calculation of the pH Value of a Solution Resulting from the Reaction Between CaCO_3_(s) and CO_2_(g)

The reaction between atmospheric CO_2_ and carbonate rocks is a key process that determines the composition of surface waters. In the case of the common minerals calcite or aragonite, the reaction reads as follows [12]:

CO_2(g)_ + H_2_O_(l)_ + CaCO_3(s)_ → Ca^2+^_(aq)_ + 2 HCO_3_^−^_(aq)_ (29)

Assume c_T_ as the total concentration of Ca^2+^ ([Ca^2+^] = c_T_). From Equation (29), and considering that HCO_3_^−^ will also be transformed into H_2_CO_3_* and CO_3_^2−^, one gets the following:

2 c_T_ = 2 [Ca^2+^] = [H_2_CO_3_*] + [HCO_3_^−^] + [CO_3_^2−^] (30)

The charge balance of this solution is:

2 [Ca^2+^] + [H_3_O^+^] = [HCO_3_^−^] + 2 [CO_3_^2−^] + [OH^−^] (31)

As far as the boundary conditions are concerned, one has the usual extremes: (i) c_T_ → 0, and (ii) high c_T_. In the former case, there is pure water with [H_3_O^+^] = (K_w_)^½^ (pH 7). In the latter case it is [H_3_O^+^] = (K_a1_* K_a2_)^1/2^, which is the pH value of a solution of bicarbonate when its concentration is high enough (pH 8.3). Consequently, the system is expected to show a limited range of pH values (7 ≤ pH ≤ 8.3, see also Figure 1b) [7,9].

That said, by substituting Equation (30) into Equation (31) and rearranging, the charge balance gets modified as follows:

[H_2_CO_3_*] + [H_3_O^+^] = [CO_3_^2−^] + [OH^−^] (32)

Use of the species fractions transforms Equation (32) into Equation (33):

(33) $$\frac{{{[H}_{3}O^{+}]}^{2}}{{{[H}_{3}O^{+}]}^{2}+K_{a1}^{*}{[H}_{3}O^{+}]+K_{a1}^{*}K_{a2}}{2c}_{T}+{[H}_{3}O^{+}]=\frac{K_{a1}^{*}K_{a2}}{{{[H}_{3}O^{+}]}^{2}+K_{a1}^{*}{[H}_{3}O^{+}]+K_{a1}^{*}K_{a2}}{2c}_{T}+\frac{K_{w}}{{[H}_{3}O^{+}]}$$

For 7 ≤ pH ≤ 8.3 (see the boundary conditions above), [HCO_3_^−^] » [CO_3_^2−^] (indeed, [HCO_3_^−^] > 10^2^ [CO_3_^2−^]). In the denominator terms, K_a1_* [H_3_O^+^] ∝ [HCO_3_^−^] and K_a1_* K_a2_ ∝ [CO_3_^2−^]; thus, the condition [HCO_3_^−^] » [CO_3_^2−^] implies that K_a1_* [H_3_O^+^] » K_a1_* K_a2_. As a consequence of this approximation, Equation (33) simplifies into Equation (34):

(34) $$\frac{{{[H}_{3}O^{+}]}^{2}}{{{[H}_{3}O^{+}]}^{2}+K_{a1}^{*}{[H}_{3}O^{+}]}{2c}_{T}+{[H}_{3}O^{+}]=\frac{K_{a1}^{*}K_{a2}}{{{[H}_{3}O^{+}]}^{2}+K_{a1}^{*}{[H}_{3}O^{+}]}{2c}_{T}+\frac{K_{w}}{{[H}_{3}O^{+}]}$$

Equation (34) is a third-order equation that can be expressed in explicit form as follows:

[H_3_O^+^]^3^ + (K_a1_* + 2 c_T_) [H_3_O^+^]^2^ − K_w_ [H_3_O^+^] − 2 c_T_ K_a1_* K_a2_ − K_a1_* K_w_ = 0 (35)

This is actually a near-exact solution, considering that the approximation K_a1_* [H_3_O^+^] » K_a1_* K_a2_ entails practically no loss in accuracy.

For 7 ≤ pH ≤ 8.3 one has [HCO_3_^−^] > [H_2_CO_3_*], although it is not always true that [HCO_3_^−^] » [H_2_CO_3_*]. Therefore, neglecting the [H_3_O^+^]^2^ terms in the denominators of Equation (35) introduces an approximation that can be assessed, by comparing the predictions of the approximate solution with those of the (near-)exact one. This simplification results in one of the simplest forms of second-order equations:

(36) $$\frac{{{[H}_{3}O^{+}]}^{2}}{K_{a1}^{*}{[H}_{3}O^{+}]}{2c}_{T}+{[H}_{3}O^{+}]=\frac{K_{a1}^{*}K_{a2}}{K_{a1}^{*}{[H}_{3}O^{+}]}{2c}_{T}+\frac{K_{w}}{{[H}_{3}O^{+}]}$$

(2 c_T_ + K_a1_*) [H_3_O^+^]^2^ = 2 c_T_ K_a1_* K_a2_ + K_a1_* K_w_ (37)

(38) $${[H}_{3}O^{+}]=\sqrt{\frac{2\;c_{T}K_{a1}^{*}K_{a2}+K_{a1}^{*}K_{w}}{2\;c_{T}+K_{a1}^{*}}}$$

**Figure 3:**
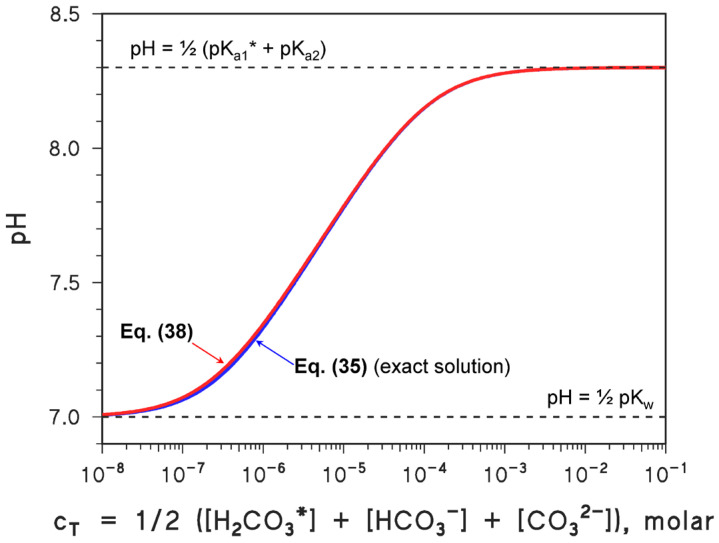
Plots (pH vs. c_T_) of the exact (Equation (35)) and approximate (Equation (38)) solutions for a system containing Ca^2+^ + HCO_3_^−^ (deriving from CaCO_3 (s)_ + CO_2 (g)_). Note the excellent agreement. The boundary conditions ([H_3_O^+^] = (K_w_)^½^ for c_T_ → 0, [H_3_O^+^] = (K_a1_* K_a2_)^1/2^ for high c_T_) are also reported on the plot.

A comparison between the near-exact Equation (35) and approximate Equation (38) solutions is provided in Figure 3, which shows that there is almost always perfect agreement between them. The only range of conditions where the approximate solution slightly diverges from the exact one is 7 < pH < 7.5, where the approximation K_a1_* [H_3_O^+^] » [H_3_O^+^]^2^ (i.e., [HCO_3_^−^] » [H_2_CO_3_*]) is not totally rigorous.

### 2.3. The Most General Case: A H_2_CO_3_* Solution (c_T_) in the Presence of Alkalinity, [Alk]

Thus far, relatively simple systems containing either H_2_CO_3_* or Ca^2+^ + HCO_3_^−^ have been taken into account. In general, however, the carbonate species H_2_CO_3_*, HCO_3_^−^, and CO_3_^2−^ occur in water in the presence of other cations and anions (e.g., NO_3_^−^, SO_4_^2−^, Cl^−^, F^−^, Na^+^, K^+^, Mg^2+^, and NH_4_^+^). Moreover, the ions H_3_O^+^ and OH^−^ always occur in an aqueous solution. Indeed, the charge balance depicted in Equation (15) represents the simplest case for a solution containing H_2_CO_3_*. The presence of additional ions is summarized by the alkalinity ([Alk]), a parameter that represents the algebraic sum of cations and anions concentrations, weighted by their respective charges (e.g., [Alk] ~2 [Ca^2+^] + 2 [Mg^2+^] + [Na^+^] + [K^+^] + [NH_4_^+^] − [Cl^−^] − [NO_3_^−^] − 2 [SO_4_^2−^]) [7,9]:

[Alk] + [H_3_O^+^] = [HCO_3_^−^] + 2 [CO_3_^2−^] + [OH^−^] (39)

By introducing Equations (9), (12) and (13) into Equation (39) and grouping the variables, the following 4th-order equation is obtained:

[H_3_O^+^]^4^ + ([Alk] + K_a1_*) [H_3_O^+^]^3^ + (K_a1_* K_a2_ + [Alk] K_a1_* − c_T_ K_a1_* − K_w_) [H_3_O^+^]^2^ + K_a1_* ([Alk] K_a2_ − 2 c_T_ K_a2_ − K_w_) [H_3_O^+^] − K_a1_* K_a2_ K_w_ = 0 (40)

**Figure 4:**
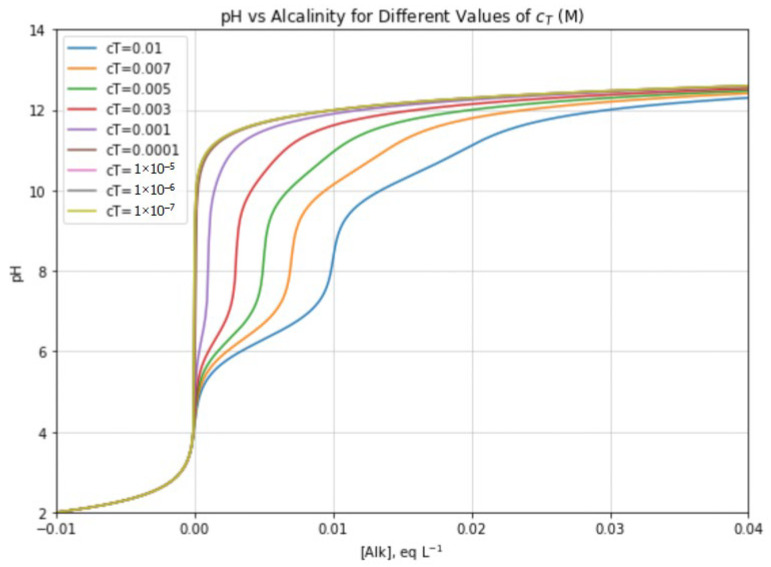
Trends of pH vs. alkalinity ([Alk]) for different values of c_T_ (exact solution, Equation (40)). The trend at low c_T_ closely resembles a strong acid/strong base titration, while the effect of the presence of carbonate species (and especially the increase in the number of inflection points) becomes apparent at sufficiently high c_T_ values.

The solutions of this equation are reported in Figure 4, as pH versus [Alk] for different values of c_T_. Interestingly, for low c_T_ values, the function appears as a strong acid−strong base titration curve, with equivalence point at ([Alk], pH) = (0, 7). At higher c_T_ values, the trend becomes more complex and exhibits several pseudo-equivalence points. The near-vertical flexes of the pH vs. [Alk] function become near-horizontal flexes in the reverse [Alk] vs. pH function, which can be expressed as follows:

(41) $$[\mathrm{Alk}]\;=\;-10^{-\mathrm{pH}}\;+\;\frac{K_{a1}^{*}10^{-\mathrm{pH}}+{2K}_{a1}^{*}K_{a2}}{10^{-2\mathrm{pH}}+K_{a1}^{*}10^{-\mathrm{pH}}+K_{a1}^{*}K_{a2}}c_{T}+\;\frac{K_{w}}{10^{-\mathrm{pH}}}$$

The pH and [Alk] values at the inflection points can be obtained by examining the function through its first and second derivatives.

Let us assume:

*N* = K_a1_* 10^−pH^ + 2 K_a1_* K_a2_ (42)

*D* = 10^−2pH^ + K_a1_* 10^−pH^ + K_a1_* K_a2_ (43)

Differentiating with respect to pH, we obtain (note that ∂ means partial derivative):

(44) $$\frac{\partial \left[\mathrm{Alk}\right]}{\partial \mathrm{pH}}=\ln(10)\;\;10^{-\mathrm{pH}}+c_{T}\frac{\left(\ln(10\right)\;K_{a1}^{*}10^{-\mathrm{pH}})\;D-N\;\left(2\ln(10\right)\;\;10^{-2\mathrm{pH}}+\ln(10)\;K_{a1}^{*}10^{-\mathrm{pH}})}{D^{2}}+\ln(10)\;K_{w}10^{\mathrm{pH}}$$

The second derivative has the following form:

(45) $$\frac{\partial^{2}\;\left[\mathrm{Alk}\right]}{\partial {\mathrm{pH}}^{2}}=-{(\ln(10))}^{2}\;\;10^{-\mathrm{pH}}+c_{T}\frac{(N\prime \prime D-ND\prime \prime )\;D^{2}-2(N\prime D-ND\prime )DD\prime }{D^{4}}+{(\ln(10))}^{2}\;K_{w}10^{\mathrm{pH}}$$

where

*N*′ = −ln(10) K_a1_* 10^−pH^ (46)

*N*″ = [ln(10)]^2^ K_a1_* 10^−pH^ (47)

*D*′ = −2 ln(10) 10^−2pH^ − ln(10) K_a1_* 10^−pH^ (48)

*D*″ = [2 ln(10)]^2^ 10^−2pH^ + [ln(10)]^2^ K_a1_* 10^−pH^ (49)

The examination of the zeros of the second derivative, together with the trend of the first derivative, provides a complete characterization of the inflection points of the alkalinity function. The first derivative with respect to [H_3_O^+^] can be expressed as follows (where *H* = [H_3_O^+^]):

(50) $$\frac{\partial \left[\mathrm{Alk}\right]}{\partial H}=c_{T}\frac{K_{a1}^{*}\;D-(K_{a1}^{*}H+2\;K_{a1}^{*}K_{a2})\;(2H+K_{a1}^{*})}{D^{2}}-\frac{K_{w}}{H^{2}}-1$$

For any physically meaningful values of [H_3_O^+^], K_a1_*, K_w_, and c_T_ > 0, the right-hand side of Equation (50) is strictly negative, with no real zeros. Considering the transformation

(51) $$\frac{\partial \left[\mathrm{Alk}\right]}{\partial \mathrm{pH}}=-\ln(10)\cdot H\cdot \left(\frac{\partial \left[\mathrm{Alk}\right]}{\partial H}\right)$$

It follows directly that the derivative of alkalinity with respect to pH is strictly positive. This confirms that [Alk] (pH) is a monotonically increasing function of pH, while its inflection points can be identified from the zeros of the second derivative.

Table 1 summarizes the pH and the corresponding alkalinity values, computed numerically with Python, where the second derivative of the alkalinity function becomes zero for different values of c_T_. The results show that, for c_T_ < 10^−5^ M, only a single inflection point is found and the curve resembles the titration curve of a strong acid with a strong base (an increase in [Alk] can be actually interpreted as the addition of a strong base to a strong acid). For c_T_ > 5 × 10^−3^ M the second derivative exhibits five zeros, as expected for a diprotic acid with sufficiently distinct pK_a_ values. In the intermediate concentration range, two of the zeros disappear. This behavior arises because the buffering effect associated with the second acidic dissociation (HCO_3_^−^ + H_2_O ⇆ H_3_O^+^ + CO_3_^2−^) is completely masked by the contribution from water autodissociation, given the high value of pK_a2_ and the low total concentration c_T_.

**Figure 5:**
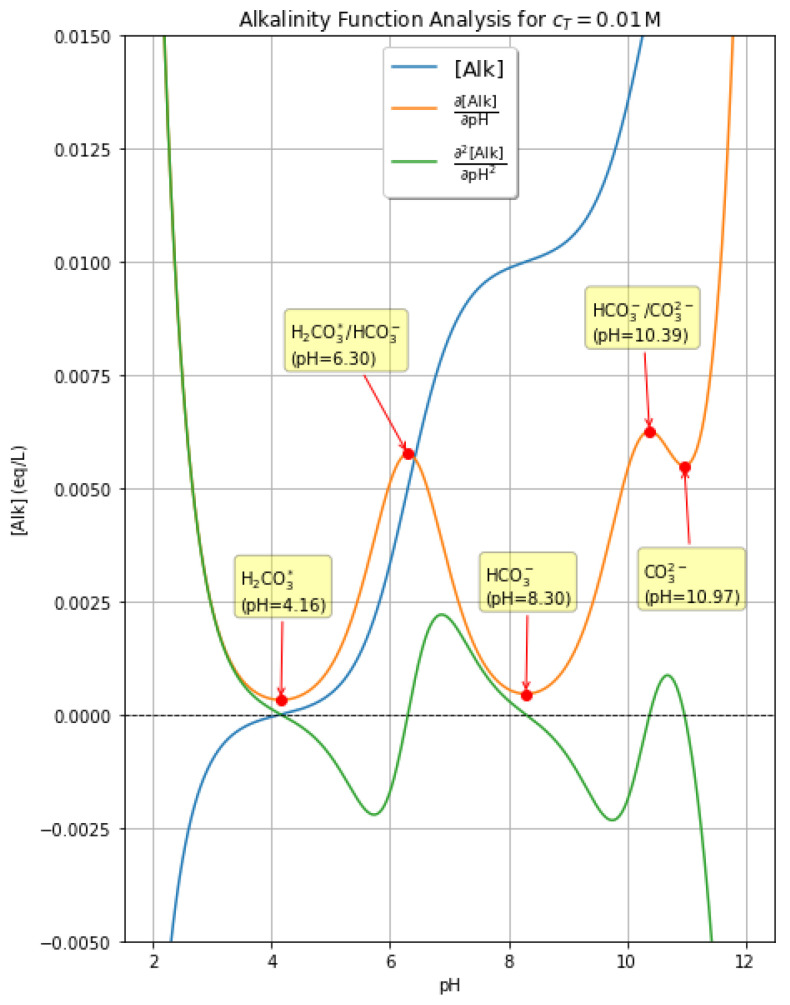
Trends of alkalinity ([Alk]) vs. pH (it is the reverse function as that shown in Figure 4), as well as of its first and second derivatives with respect to pH. The chemical composition of the solution at inflection points is highlighted.

To better illustrate the behavior of the system, we focus on the most complete case, where the second derivative displays five zeros, as it is the case with c_T_ = 0.01 M as shown in Figure 5. In this panel, the zeros of the second derivative are highlighted, which correspond to maxima or minima of the first derivative, and labels are assigned to highlight the predominant species under these conditions.

The first derivative of the function, $\frac{\partial [\mathrm{Alk}]}{\partial \mathrm{pH}}$ vs. pH (Equation (44)), provides a measure of the buffering power of the solution. High values of $\frac{\partial [\mathrm{Alk}]}{\partial \mathrm{pH}}$ indicate that pH varies very little for a given variation in alkalinity, whereas low values correspond to higher pH sensitivity. The function $\frac{\partial [\mathrm{Alk}]}{\partial \mathrm{pH}}$ vs. pH for c_T_ = 0.01 mol L^−1^ has the trend shown in Figure 5, which is consistent with the flexes of the function [Alk] vs. pH that are observed just above pH 4, just above pH 8, and around pH 11. As shown in Figure 5, the buffering power is understandably very high in strongly acidic and strongly basic conditions, where the presence of high concentrations of strong acids or bases is sufficient to limit the pH variations. The buffering power is also high in the presence of the acid/base couples H_2_CO_3_*/HCO_3_^−^ (pH = pK_a1_*) and HCO_3_^−^/CO_3_^2−^ (pH = pK_a2_), while it is understandably minimum for the amphoteric species HCO_3_^−^ (pH = ½ (pK_a1_* + pK_a2_)). Finally, further minima in the buffer capacity are observed for [Alk] = 0 (pH 7 for c_T_ → 0, pH ~4 for c_T_ = 0.01 M, where the absence of strong acids or bases limits the capacity of the system to buffer pH variations), and in the presence of CO_3_^2−^ alone (pH ~11).

**Figure 6:**
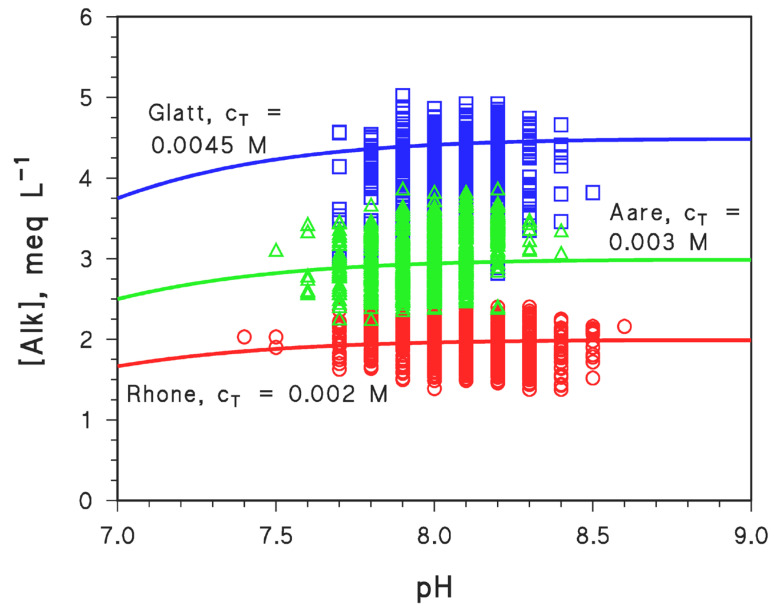
Values of alkalinity ([Alk]) and pH measured in Swiss rivers (☐: Glatt at Rheinsfelden, sampled in the period 1976–2011; ∆: Aare at Brugg, 1974–2011; ◯: Rhône at Chancy, 1977–2011) [1]. The solid curves represent the predictions made with Equation (41) and suitable values of c_T_.

A comparison between the predictions of Equation (41) and actual field data of water alkalinity and pH is reported in Figure 6, which refers to water samples collected in Swiss rivers (Aare, Rhône, and Glatt) in the time period ranging from the 1970s to 2010s [1]. It can be seen from the figure that Equation (41) (represented by solid curves) is well within the range of the measured field values, with [Alk] being reasonably predicted from the pH values within ±0.5 (Rhône) or ±1 (Glatt) meq L^−1^ units.

Another interesting issue is that the total concentration of carbonate species is c_T_ ≈ [Alk] (see Figure 6), which is often observed at the typical pH values of natural waters and is also expected from the charge balance (see Equation (39)), considering the usually limited contribution by [H_3_O^+^] and [OH^−^] in ~neutral conditions and given that [HCO_3_^−^] » [CO_3_^2−^] [7,9]. With these assumptions, Equation (39) transforms into [Alk] ≈ [HCO_3_^−^] ≈ c_T_. Moreover, comparison with field data suggests that Equation (41) can reasonably describe the relationship between [Alk] and pH in natural surface waters.

## 3. Methods

In this work, we have used analytical rather than thermodynamic K_a_ values. This is equivalent to considering that the ionic strength *I* is not significantly affected by the concentrations of H_2_CO_3_* (c_T_), which provides the ions carbonate and bicarbonate to the solution.

If thermodynamic K_a_ values were to be taken into account, the charge balance Equation (15) would be modified into Equation (52) within the framework of the Davies approximation, where *I* = ½ Σ_i_(z_i_^2^ [i]) is the ionic strength of the solution, i represents a generic species having charge z_i_, either positive or negative, and {} denotes an activity value [7,9]:

(52) $$\begin{array}{c} \left\{H_{3}O^{+}\;\}(10^{0.51\left(\frac{\sqrt{I}}{1+\sqrt{I}}-0.30I\right)})=\{{\mathrm{HCO}}_{3}^{-}\}\;(10^{0.51\left(\frac{\sqrt{I}}{1+\sqrt{I}}-0.30I\right)}\right)\\ +2\;\{{{\mathrm{CO}}_{3}}^{2-}\}\;(10^{2.04\left(\frac{\sqrt{I}}{1+\sqrt{I}}-0.30I\right)})+\{{\mathrm{OH}}^{-}\}\;(10^{0.51\left(\frac{\sqrt{I}}{1+\sqrt{I}}-0.30I\right)}) \end{array}$$

A major disadvantage of Equation (52) is that the exact value of *I* is usually not known at the beginning because it depends on equilibrium calculation results. Therefore, recurring calculations till convergence are typically required.

By following our alternative approach, all calculations based on analytical K_a_ values here were performed in Python 3.11, using the packages ‘NumPy’, ‘SciPy’, ‘Matplotlib’ (for plotting), and ‘pandas’ (for data export) [8,11]. Four different scripts were developed:

1. The first script solves different simplified equilibrium equations for [H_3_O^+^] in carbonic acid systems. Numerical solutions were obtained with ‘fsolve’, and the corresponding pH values were compared across five approximations. The results are reported in Figure 2.


import numpy as np
import matplotlib.pyplot as plt
from scipy.optimize import fsolve
Ka1_star = 10**-6.3
Kw = 1e-14
cT = np.logspace(-8, -1, 500)
pH_eq1 = []  
pH_eq2 = []  
pH_eq3 = []  
pH_eq4 = []  
pH_eq5 = []  
def eq1(H, cT_val):
    return H - (Ka1_star * cT_val) / (H + Ka1_star) - Kw / H
def eq2(H, cT_val):
    return H - cT_val - Kw / H
def eq4(H, cT_val):
    return H - (Ka1_star * cT_val) / (H + Ka1_star)
for ct in cT:    
    H1 = fsolve(eq1, ct, args=(ct))[0]
    pH_eq1.append(-np.log10(H1))  
    H2 = fsolve(eq2, ct, args=(ct))[0]
    pH_eq2.append(-np.log10(H2)) 
    H3 = np.sqrt(Ka1_star * ct + Kw)
    pH_eq3.append(-np.log10(H3))    
    H4 = fsolve(eq4, ct, args=(ct))[0]
    pH_eq4.append(-np.log10(H4))    
    H5 = np.sqrt(Ka1_star * ct)
    pH_eq5.append(-np.log10(H5))
plt.figure(figsize=(10, 6))
plt.semilogx(cT, pH_eq1, '-', label='[H⁺] = (Ka1·cT)/(H⁺ + Ka1) + Kw/H⁺')
plt.semilogx(cT, pH_eq2, '--', label='[H⁺] = cT + Kw/H⁺')
plt.semilogx(cT, pH_eq3, ':', label='[H⁺] = √(Ka1·cT + Kw)')
plt.semilogx(cT, pH_eq4, '-.', label='[H⁺] = (Ka1·cT)/(H⁺ + Ka1)')
plt.semilogx(cT, pH_eq5, '-', label='[H⁺] = √(Ka1·cT)')
plt.xlabel('cT [mol/L]')
plt.ylabel('pH')
plt.title('Comparison of five approximations for [H⁺] in H₂CO₃')
plt.legend()
plt.grid(False)  
plt.tight_layout()
plt.show()

2. The second script derives and solves a cubic equation for [H_3_O^+^], representing an approximate description of the carbonate system. The cubic equation was solved numerically with ‘fsolve’, while an analytical approximation was also implemented. The two solutions (exact vs. approximate) were then compared across a range of c_T_ values (Figure 3):


import numpy as np
import matplotlib.pyplot as plt
from scipy.optimize import fsolve
Ka1 = 10**-6.3   
Ka2 = 10**-10.3  
Kw = 1e-14     

cT_values = np.logspace(-8, -1, 500)
pH_exact = []
pH_approx = []
def cubic_eq(H, cT_val):
    return H**3 + (Ka1 + 2*cT_val)*H**2 - Kw*H - Ka1*(2*cT_val*Ka2 + Kw)
def approx_H(cT_val):
    numerator = Ka1 * (2*cT_val*Ka2 + Kw)
    denominator = 2*cT_val + Ka1
    return np.sqrt(numerator / denominator)
for ct in cT_values:
    H_root = fsolve(cubic_eq, 1e-7, args=(ct))[0]     
    H_approx = approx_H(ct)                            
    pH_exact.append(-np.log10(H_root))
    pH_approx.append(-np.log10(H_approx))
plt.figure(figsize=(10, 6))
plt.semilogx(cT_values, pH_exact, label=r'exact pH: $[H^+]^3 + (K_{a1}* + 2c_T)[H^+]^2 - K_w[H^+] - K_{a1}*(2c_T K_{a2} + K_w) = 0$')
plt.semilogx(cT_values, pH_approx, '--', label=r'approximate pH: $[H^+] = \sqrt{\frac{K_{a1}*(2c_T K_{a2} + K_w)}{2c_T + K_{a1}*}}$')
plt.xlabel('cT [mol/L]')
plt.ylabel('pH')
plt.title('pH of a bicarbonate solution')
plt.legend()
plt.grid(False)
plt.tight_layout()
plt.show()

3. The third script computes the exact relationship between alkalinity ([Alk]) and pH for different values of the total inorganic carbon concentration (c_T_). The hydrogen ion concentration was obtained by solving the fourth-degree equation with the Brent’s root-finding method (‘brentq’). This allowed a robust identification of all valid physical roots. The results were plotted as pH vs. alkalinity curves, for different c_T_ values (Figure 4).


import numpy as np
import matplotlib.pyplot as plt
from scipy.optimize import brentq
import pandas as pd
Ka1 = 10**-6.3
Ka2 = 10**-10.3
Kw  = 1e-14
Alk_values = np.linspace(-0.01, 0.04, 400)
H_min, H_max = 1e-14, 1.0
N_scan       = 400
xtol_brentq  = 1e-15
rtol_brentq  = 1e-12
cT_values = [1e-2, 0.007, 0.005, 0.003, 1e-3, 1e-4, 1e-5, 1e-6, 1e-7]
def fH(H, Alk, Ka1, Ka2, Kw, cT):
    return (H**4 
            + H**3*(Alk + Ka1) 
            + H**2*(Alk*Ka1 + Ka1*Ka2 - Ka1*cT - Kw) 
            + H*(Alk*Ka1*Ka2 - 2*Ka1*Ka2*cT - Ka1*Kw) 
            - Ka1*Ka2*Kw)
def find_sign_change_brackets(Alk, cT):
    Hgrid = np.logspace(np.log10(H_min), np.log10(H_max), N_scan)
    fvals = fH(Hgrid, Alk, Ka1, Ka2, Kw, cT)
    brackets = []
    for i in range(len(Hgrid) - 1):
        f1, f2 = fvals[i], fvals[i+1]
        if np.isnan(f1) or np.isnan(f2): continue
if abs(f1) < 1e-300: f1 = np.copysign(1e-300, f1 if f1 != 0 else 1.0)
if abs(f2) < 1e-300: f2 = np.copysign(1e-300, f2 if f2 != 0 else -1.0)
        if f1 * f2 < 0.0:
            brackets.append((Hgrid[i], Hgrid[i+1]))
            return brackets
def roots_with_brentq(Alk, cT):
    brackets = find_sign_change_brackets(Alk, cT)
    roots = []
    for (a, b) in brackets:
        try:
            r = brentq(fH, a, b, args=(Alk, Ka1, Ka2, Kw, cT),
               xtol=xtol_brentq, rtol=rtol_brentq, maxiter=200)
            if H_min <= r <= H_max:
                roots.append(r)
              except ValueError:
              pass
              return sorted(set(roots))
plt.figure(figsize=(8, 6))
all_results = {"Alk": Alk_values}
for cT in cT_values:
    H_list, pH_list = [], []
    prev_H = None
    for i, Alk in enumerate(Alk_values):
      roots = roots_with_brentq(Alk, cT)
    if not roots:
      H_list.append(np.nan)
      pH_list.append(np.nan)
      prev_H = None
      continue
    if i == 0:
      h_choice = max(roots)  
    else:
      log_prev = np.log(prev_H) if prev_H else 0
      h_choice = min(roots, key=lambda h: abs(np.log(h) - log_prev))
      prev_H = h_choice
      H_list.append(h_choice)
      pH_list.append(-np.log10(h_choice))
      all_results[f"pH_cT={cT:g}"] = pH_list
      plt.plot(Alk_values, pH_list, label=f"cT={cT:g}")
plt.xlabel(r"[Alk], eq L$^{-1}$")
plt.ylabel("pH")
plt.title("pH vs Alcalinity for Different Values of $c_T$ (M)")
plt.xlim(-0.01, 0.04)
plt.ylim(2, 14)
plt.grid(True, linestyle="-", alpha=0.6)
plt.legend()
plt.tight_layout()
plt.show()
df = pd.DataFrame(all_results)
df.to_excel("pH_vs_Alk.xlsx", index=False)
print("File Excel saved as pH_vs_Alk.xlsx")

4. In addition to the equilibrium and alkalinity solvers, a dedicated Python script was developed to compute the first and second derivatives of the alkalinity function, with respect to both H_3_O^+^ concentration and pH. The code was implemented using ‘NumPy’ for efficient vectorized calculations and ‘Matplotlib’ for visualization (Figure 5). Symbolic expressions for the derivatives were derived analytically and then translated into Python functions. The script evaluates the alkalinity, expressed as a function of pH, its first derivative ∂[Alk]/∂pH, and the second derivative ∂^2^[Alk]/∂pH^2^ over a dense grid of pH values. Zeros of the second derivative, corresponding to inflection points of the alkalinity curve, were identified numerically and compared across different conditions (Table 1).


import numpy as np
import matplotlib.pyplot as plt
from scipy.optimize import brentq
Ka1 = 10**-6.3
Ka2 = 10**-10.3
Kw  = 1e-14     
cT  = 1e-2  
pH = np.linspace(2, 12, 1000)
H = 10**(-pH)
D = H**2 + Ka1*H + Ka1*Ka2
Alk = cT * ((Ka1*H)/D + (2*Ka1*Ka2)/D) + (Kw/H) - H
dAlk_dH = (
    cT * (Ka1*D - (Ka1*H + 2*Ka1*Ka2)*(2*H + Ka1)) / D**2
    - Kw/H**2 - 1)
z = -np.log(10) * H * dAlk_dH   
d2Alk_dH2 = (2*Kw/H**3 - cT*Ka1 * ((2*H + 4*Ka2)*D - 2*(H**2 + 4*Ka2*H + Ka1*Ka2)*(2*H + Ka1)) / D**3)
y = -np.log(10) * (H * dAlk_dH + H**2 * d2Alk_dH2)   
zeros = []
for i in range(len(pH)-1):
    if y[i] == 0:
        zeros.append(pH[i])
    elif y[i]*y[i+1] < 0:  
        try:
       root = brentq(lambda xx: np.interp(xx, pH, y), pH[i], pH[i+1])
       zeros.append(root)
       except ValueError:
       pass
print("Zeros of the second derivative (pH values corresponding to the minima and maxima of the first derivative):")
print(zeros)
labels = [
    r"$\mathrm{H_2CO_3^*}$",
    r"$\mathrm{H_2CO_3^*/HCO_3^-}$",
    r"$\mathrm{HCO_3^-}$",
    r"$\mathrm{HCO_3^-/CO_3^{2-}}$",
    r"$\mathrm{CO_3^{2-}}$"]
plt.figure(figsize=(7,10))
plt.plot(pH, Alk, label=r"$[\mathrm{Alk}]$")
plt.plot(pH, z, label=r"$\frac{\partial [\mathrm{Alk}]}{\partial \mathrm{pH}}$")
plt.plot(pH, y, label=r"$\frac{\partial^2 [\mathrm{Alk}]}{\partial \mathrm{pH}^2}$")
plt.axhline(0, color="k", linestyle="--", linewidth=0.8)
for i, root in enumerate(zeros):
    z_val = np.interp(root, pH, z)
    plt.plot(root, z_val, "ro")  # punto rosso
    label = labels[i] if i < len(labels) else labels[-1]
    offset_x, offset_y = -0.7, 0.002
    if i == len(zeros) - 1:
        offset_x, offset_y = -0.7, -0.003 # sinistra e più in basso
    if i == len(zeros) - 4:
        offset_x, offset_y = -2, 0.002
    if i == len(zeros) - 2:
        offset_x, offset_y = -1, 0.002
    plt.annotate(
        f"{label}\n(pH={root:.2f})",
        xy=(root, z_val),
        xytext=(root + offset_x, z_val + offset_y),
        arrowprops=dict(arrowstyle="->", color="red"),
        fontsize=10,
        bbox=dict(boxstyle="round,pad=0.3", fc="yellow", alpha=0.3))
plt.xlabel("pH")
plt.ylabel(r"$[\mathrm{Alk}] \; (\mathrm{eq/L})$")
plt.ylim(-0.005, 0.015)
plt.title(r"Alkalinity Function Analysis for $c_T = 0.01 \,\mathrm{M}$")
plt.legend(fontsize=13, loc="best", frameon=True, fancybox=True, shadow=True)
plt.grid(True)
plt.show()


## 4. Conclusions

In this work, we discuss several strategies that can be used to calculate the equilibrium pH values of solutions containing inorganic carbon species. In the cases of H_2_CO_3_* and of Ca^2+^ + HCO_3_^−^, we show that one or several approximations can be introduced in different ranges of carbonate concentration (c_T_) and pH.

As far as H_2_CO_3_* is concerned, typical approximations involve neglecting the self-ionization of water and/or assuming that carbonic acid is extensively or poorly dissociated. However, neglecting the self-ionization of water returns a solution that fails to meet the boundary condition foreseeing pH ~ ½ pK_w_ for c_T_ → 0. On the other hand, the assumption that carbonic acid is extensively dissociated predicts the behavior of the system well at low c_T_, but it fails for relatively high c_T_ values (>10^−7^ M). We obtained the most suitable approximation by assuming that carbonic acid is poorly dissociated, but without neglecting water self-ionization. The first assumption works well at high c_T_, while the latter satisfies the boundary condition for c_T_ → 0. Interestingly, the best approximation in the case of H_2_CO_3_* was that fully satisfying both boundary conditions.

In the case of Ca^2+^ + HCO_3_^−^, a suitable approximation implied that bicarbonate is the main species of inorganic carbon, which prevails over both carbonic acid and carbonate. This approximation showed reasonable validity in a wide set of conditions, and we show that it also satisfied the boundary conditions of the system. From both cases, it can thus be derived that the best approximate solution should be chosen by taking into account the behavior of the system for c_T_ → 0 and for high c_T_ values (the latter is the chemical equivalent of the mathematical condition “c_T_ → ∞”). If the approximation meets these conditions (e.g., by not neglecting [OH^−^] in the charge balance if $\underset{c_{T}\to 0}{\lim}$(pH) = ½ pK_w_), one can be reasonably confident that it will also describe the system well enough in intermediate cases.

We also dealt with the more general case of H_2_CO_3_* combined with alkalinity, where, however, the wide range of possible pH values prevented any meaningful approximation to be attempted. In that case, only the exact solution was appropriate. The Python code to handle such solution and to study its pH behavior is provided here to help readers with a system that is definitely less manageable than a second-order equation. At the same time, however, such an exact solution proved suitable to reproduce the field data of [Alk] vs. pH in the case of Swiss rivers, which is an argument in favor of the general validity of that approach.

Overall, our calculations describe several systems containing H_2_CO_3_*, by combining equilibrium relationships with charge and mass balances. The latter allowed for analytical expressions to be derived, which could then be solved either analytically or numerically. The advantage of this approach is that the dependencies of pH on c_T_ and/or [Alk] are always made explicit here, in contrast to the purely numerical outputs produced by dedicated pH-calculation software packages. Therefore, our approaches should facilitate the mathematical description of aqueous systems containing H_2_CO_3_*.

## Figures and Tables

**Table 1:** Flexes of the pH vs. [Alk] function, for different values of c_T_. These flexes have been identified as the zeros of the second-derivative of [Alk] vs. pH (see also Figure 5).

c_T_ [M]	pH	Alk [eq/L]
1.0 × 10^−2^	10.973	1.918 × 10^−2^
	10.385	1.573 × 10^−2^
	8.295	1.000 × 10^−2^
	6.300	5.002 × 10^−3^
	4.156	1.518 × 10^−6^
7.0 × 10^−3^	10.826	1.306 × 10^−2^
	10.442	1.134 × 10^−2^
	8.293	6.999 × 10^−3^
	6.300	3.501 × 10^−3^
	4.235	1.521 × 10^−6^
5.0 × 10^−3^	8.291	5.000 × 10^−3^
	6.300	2.500 × 10^−3^
	4.309	1.524 × 10^−6^
1.0 × 10^−3^	8.258	9.999 × 10^−4^
	6.299	4.989 × 10^−4^
	4.670	1.551 × 10^−6^
1.0 × 10^−4^	8.049	9.992 × 10^−5^
	6.283	4.852 × 10^−5^
	5.221	1.685 × 10^−6^
1.0 × 10^−5^	7.610	9.937 × 10^−6^
1.0 × 10^−6^	7.168	9.607 × 10^−7^
1.0 × 10^−7^	7.020	9.313 × 10^−8^

## Data Availability

The raw data supporting the conclusions of this article will be made available by the authors on request.
